# Regulation of GacA in *Pseudomonas chlororaphis* Strains Shows a Niche Specificity

**DOI:** 10.1371/journal.pone.0137553

**Published:** 2015-09-17

**Authors:** Jun Li, Yang Yang, Jean-Frédéric Dubern, Hui Li, Nigel Halliday, Leonid Chernin, Kexiang Gao, Miguel Cámara, Xiaoguang Liu

**Affiliations:** 1 School of Food and Biological Engineering, Jiangsu University, Zhenjiang, 212013, China; 2 Institute of Life Sciences, Jiangsu University, Zhenjiang, 212013, China; 3 School of Life Sciences, Centre for Biomolecular Sciences, University of Nottingham, Nottingham, NG7 2RD, United Kingdom; 4 Department of Plant Pathology, Shandong Agricultural University, Taian, 271018, China; 5 Department of Plant Pathology and Microbiology, The Robert H. Smith Faculty of Agriculture, Food and Environment, The Hebrew University of Jerusalem, Rehovot, 76100, Israel; Chang-Gung University, TAIWAN

## Abstract

The GacS/GacA two-component system plays a central role in the regulation of a broad range of biological functions in many bacteria. In the biocontrol organism *Pseudomonas chlororaphis*, the Gac system has been shown to positively control quorum sensing, biofilm formation, and phenazine production, but has an overall negative impact on motility. These studies have been performed with strains originated from the rhizosphere predominantly. To investigate the level of conservation between the GacA regulation of biocontrol-related traits in *P*. *chlororaphis* isolates from different habitats, the studies presented here focused on the endophytic isolate G5 of *P*. *chlororaphis* subsp. *aurantiaca*. A *gacA* mutant deficient in the production of *N*-acylhomoserine lactones (AHLs) and phenazine was isolated through transposon mutagenesis. Further phenotypic characterization revealed that in strain G5, similar to other *P*. *chlororaphis* strains, a *gacA* mutation caused inability to produce biocontrol factors such as phenazine, HCN and proteases responsible for antifungal activity, but overproduced siderophores. LC-MS/MS analysis revealed that AHL production was also practically abolished in this mutant. However, the wild type exhibited an extremely diverse AHL pattern which has never been identified in *P*. *chlororaphis*. In contrast to other isolates of this organism, GacA in strain G5 was shown to negatively regulate biofilm formation and oxidative stress response whilst positively regulating cell motility and biosynthesis of indole-3-acetic acid (IAA). To gain a better understanding of the overall impact of GacA in G5, a comparative proteomic analysis was performed revealing that, in addition to some of the traits like phenazine mentioned above, GacA also negatively regulated lipopolysaccharide (LPS) and trehalose biosynthesis whilst having a positive impact on energy metabolism, an effect not previously described in *P*. *chlororaphis*. Consequently, GacA regulation shows a differential strain dependency which is likely to be in line with their niche of origin.

## Introduction

Rhizospheric or endophytic *Pseudomonas* species such as *P*. *chlororaphis*, *P*. *protegens* and *P*. *fluorescens* have been isolated and identified from numerous plant species displaying a broad spectrum of antagonistic activity against phytopathogenic fungi, bacteria and nematodes, and plant growth-promoting potential due to the production of an array of secondary metabolites including antibiotics such as 2,4-diacetylphloroglucinol, pyrrolnitrin, pyoluteorin, hydrogen cyanide (HCN), phenazines, and the plant auxin indole-3-acetic acid (IAA), volatile organic compounds like acetoin and 2R, 3R-butanediol, as well as extracellular chitinases and proteases[1, 2]. Thereby, plant-beneficial *Pseudomonas* species are key components of the ecological processes that suppress plant pathogens and promote plant growth in agricultural and natural environments and several strains within the *P*. *fluorescens* group are used commercially to manage plant diseases [3].

The precise regulation of gene expression in response to various environmental stimuli and the cellular physiological state is essential for the survival of bacterial populations in different habitats and their biological control activity against plant diseases [4]. Many of these genes are known to be coordinately regulated by quorum sensing system at a transcriptional level mediated by *N*-acyl-homoserine lactone (AHL) signals in biocontrol bacteria [5,6]. In addition, numerous two-component systems (TCSs) act as key mediators of successful adaptation to changing environments in bacteria. For example, the GacS/GacA system consisting of the sensor kinase GacS and the response regulator GacA which is highly conserved among pseudomonads and other γ-proteobacteria operate a switch between primary and secondary metabolism [7]. The Gac/Rsm signal transduction pathway controls gene expression at both transcriptional and post-transcriptional levels, which is mediated largely through activation of the transcription of small RNAs (sRNAs) such as CsrB/C or RsmX/Y/Z. These CsrB family sRNAs sequester the RsmA/CsrA family proteins binding to the Shine-Dalgarno (SD) sequences, thereby relieve translational repression [7, 8]. The range of phenotypes modulated by the Gac/Rsm cascade typically involves management of carbon storage, regulation of the production of AHL signals, motility and biofilm formation, virulence determinants and biocontrol factors. However, the GacS/GacA system has known to differentially control the accessory genome across bacterial genera displaying the remarkable versatility [7, 9].

Apart from quorum sensing circuits, many global regulators such as two sensor kinases LadS and RetS [10], and the stationary-phase sigma factor RpoS [11] have been reported as parts of the Gac/Rsm cascade network in pseudomonads. Recent studies demonstrated that Lon protease negatively affects GacA protein stability and expression in *P*. *(fluorescens) protegens* CHA0[12]. Increasing genome-wide transcriptomic evidence demonstrated that GacA influenced 10% to 15% transcript levels in the genome of pseudomonads including numerous transcriptional regulatory genes. These findings indicated a central role of the Gac system in the complex regulatory network, and the wider impact of GacA on the transcriptome profiling likely to be mediated via intermediate transcriptional regulators including quorum sensing circuits in pseudomonads [13–14]. However, proteomic studies on the role of the Gac system in posttranscriptional regulation of gene expression in pseudomonads remains less documented. For example, recent comparative proteomic analysis between the rhizobacterium *P*. *chlororaphis* O6 and a *gacS* mutant was performed showing the differentially expressed proteins function in secondary metabolism, oxidative stress, cell signaling and secretion [15].

The endophytic microorganisms live within host plants for at least part of their life cycle without causing apparent disease symptoms, and colonize ecological niches similar to that of phytopathogens, thereby play an important role in the development of environmentally-friendly methods to manage plant diseases [2, 16]. *Pseudomonas chlororaphis* subsp. *aurantiaca* G5 is an endophytic strain isolated from the stems of Chinese parsley (*Coriandrum sativum* L.) in Taian, China [16] which is different from other rhizobacteria of *P*. *chlororaphis* such as *P*. *chlororaphis* subsp. *aureofaciens* 30–84 or O6 isolated from wheat rhizosphere or soil in the USA [3, 15, 17], whereas *P*. *chlororaphis* PA23 originated from soybean root tips, Canada [18]. Similar to other *P*. *chlororaphis* strains, G5 can produce several antibiotics such as phenazine, pyrrolnitrin and hydrogen cyanide (HCN), extracellular protease, and plant hormone IAA which are responsible for biocontrol and plant growth-promoting potential, as well as high level of AHL signal molecules [3, 16]. However, the AHL profile produced by *P*. *chlororaphis* G5 has not been characterized yet. Interestingly, both phenazine and AHLs have been shown to be required for biofilm formation in closely related biocontrol strains 30–84 [17] and PA23 [18–20]. Here we isolated and selected a *gacA* mutant from a random Mini-Tn5 transposon library of strain G5. Furthermore, we provided evidence that GacA regulates a variety of biological processes in the endophytic *P*. *chlororaphis* G5 and showed that, although there is a level of conservation in their regulation which is shared with different *P*. *choloraphis* strains described in the literature, the Gac system clearly shows differential regulation of some of these traits described above, which may be in line with the niche origin of these strains. These results may help with developing and optimizing strain G5 as seed inoculants with a view to improve its biocontrol and plant growth-promoting potential.

## Materials and Methods

### Microorganisms, media and growth conditions

The bacterial strains and plasmids used in this study are listed in S1 Table. *Pseudomonas chlororaphis* subsp. *aurantiaca* G5 and the AHL biosensor strain *Chromobacterium violaceum* CV026 were grown in Luria-Bertani (LB) medium at 28°C with shaking. Whereas *Escherichia coli* strains were grown in LB at 37°C unless otherwise stated. When required, antibiotics were added at the following concentrations: ampicillin (Ap), 100μg/mL; kanamycin (Km), 50μg/mL; tetracycline (Tc), 200μg/mL and rifampicin (Rif), 30μg/mL. Bacterial growth was monitored by measuring the optical density at 600 nm. The fungal pathogen *Rhizoctonia cerealis* (authors’ collection) was routinely cultured on potato dextrose agar (PDA) (Difco) at 25°C.

### DNA preparation and manipulations

Standard methods were used for plasmid and genomic DNA isolation, restriction enzyme digestion, agarose gel electrophoresis, ligation, and transformation [21].

### Transposon mutagenesis and transposon-insertion mapping

For construction of a mini-Tn5 mutant library to select pleiotropic regulators deficient in phenazine biosynthesis and antifungal activity, a transposon random mutagenesis of *P*. *chlororaphis* G5 was carried out with the donor *E*. *coli* S17-1-λpir/pUT-miniTn5 Km as previously described [22] to select for *P*. *chlororaphis* transconjugants on LB agar plates containing rifampicin and kanamycin. White mutant colonies unable to produce orange pigment indicating loss of phenazine were picked and stored at -80°C with 25% glycerol. One of these mutants designated G5-6 also impaired in AHL signals by cross-streak against the biosensor *C*. *violaceum* CV026 and in antifungal activity against *R*. *cerealis* on PDA plates was selected for further analysis. Mapping of the insertion sites of the mini-Tn5 transposon using inverse PCR technique was performed as described previously [23]. The primer pairs invF: 5’-CCGCGTTCGTGATTGTAC-3’ and invR: 5’-TTCAGGCTGCGCAA- CTGTT-3’ were designed to the known sequence of miniTn5-Km in opposite orientations, and the PCR amplification of the regions flanking the transposon were performed using self-ligation of *Sal*I-digested chromosomal DNA obtained from the G5-6 mutant as template, followed by cloning and sequencing to map the transposon sites. The deduced amino acid sequences of the sequenced PCR fragments were compared with the protein sequence database using the BLASTX Algorithm (http://www.ncbi.nlm.nih.gov).

### Complementation of *gacA* mutant

For complementation of the mutant G5-6, using primer pairs *gacA*-pF: 5’-gcgGGATCCATACCCTTG-CCGAGCTTT-3’ (with *BamH*I underlined) and *gacA*-R: 5’-ggcAAGCTTCTGCAGGTGGAAAGAAAAG-3’ (with *Hind*III underlined), a 898-bp *BamH*I-*Hind*III PCR fragment of the *gacA* gene from strain G5 with the native promoter was ligated into the same restriction sites of the broad-host-range vector pUCP26, followed by transformed into *E*.*coli* S17-1. The resulting plasmid pUCP26-*gacA* was introduced into the *gacA* mutant G5-6 by biparental mating. The complemented strain G5-6/*gacA* ^+^ was selected on LB agar plates with kanamycin and tetracycline and was verified by recovery of orange pigment and AHLs production, and antifungal activity.

### Extraction and identification of AHLs by LC-MS/MS

All tested bacteria were grown in 10 ml LB in triplicate with shaking at 30°C for 24 hr, AHLs were extracted and examined by LC-MS/MS as described previously[6]. MS analysis was conducted under positive electrospray conditions (+ES) with the MS in MRM (multiple reaction monitoring) mode, screening the LC eluent specifically for all unsaturated, 3-oxo and 3-OH AHLs with acyl chain lengths between 4–14, and comparing LC retention time of detected peaks with synthetic standards as control.

### Assay of biocontrol-related phenotypes

Suppression of the fungal pathogen *Rhizoctonia cerealis*, the production of hydrogen cyanide (HCN) and siderophore and protease activity were determined as previously described [24]. Phenazine was tested as described by Yang et al., 2011 [25]. Briefly, bacteria were grown with shaking in 5 ml of LB broth supplemented with 0.2% glucose at 30°C for 48 hr. The culture supernatants were acidified with 45 μl of 10% trifluoroacetic acid and extracted twice with 10 ml of ethyl acetate. Organic phases were pooled and evaporated to dryness, and residues were re-dissolved in 20 μl of methanol. For TLC assay, 10 μl of each sample was loaded onto the thin-layer Silica Gel 60 F254 plate (Merck, Germany). The plate was then placed in a mixture of benzene/acetic acid (95:5 vol/vol) to develop for 1 hr. Phenazine-1-carboxylic acid (PCA) was visualized by UV irradiation at 256 nm.

To assess the plant hormone IAA biosynthesis, strain G5 and the *gacA* mutant G5-6 were incubated in triplicate in LB supplemented with 200 μg/ml tryptophan at 28°C in the dark with shaking for 48 hr. Culture supernatants were acidified to pH2.0 with 4N HCl, and extracted twice with ethyl acetate. HPLC analysis (Agilent 1200LC, USA) with a diode array detector at 280 nm using a reversed-phase C18 column (5 μm, 150 x 4.6 mm Zorbax eclipse XDB) was performed to determine IAA production with two independent experiments as previously described [24]. Synthetic IAA (50 μg/ ml, Sigma) was used as a standard.

### Cell motility and biofilm formation

For cell motility, overnight bacterial cultures were inoculated on the swimming (0.25% agar LB) or swarming (0.4% agar LB) plates in triplicates. After 24 hr incubation at room temperature, the motility diameters were measured.

Biofilm formation was evaluated using a Laser Confocal Scanning Microscopy (LCSM) as described by Müsken et al.(2010) [26] with minor modifications. Briefly, overnight bacterial cultures from single colonies were normalized to an OD_600_ = 0.05 in 0.1xLB broth, then 1ml of the diluted cultures was pipetted into each well of 24-well glass bottom Sensoplate (Greiner Bio-One, Germany) with 6 replicates, and incubated at 30°C, 80 rpm for 24 hr. After dying with diluted Syto9 (Life Technology, USA) at the final concentration of 1.0 μM in the wells, bacteria were grown for a further 24 hr. Biofilms were then washed with PBS buffer (pH 7.0) and visualized using a Zeiss LSM 510 META/AxioVert 200 Confocal Microscope. Biofilm quantification was performed using COMSTAT2 software (http://www.comstat.dk) [27].

### Superoxide dismutase activity and tolerance to oxidative stress

Determination of the superoxide dismutase (SOD) activity was performed using the Superoxide Dismutase Assay Kit (Suzhou Comin Biotech, Ltd., China) according to manufacturer’s recommendations [28]. One unit of SOD activity was defined as the amount required to inhibit the rate of reduction of superoxide anion by 50% per microgram of protein in the xanthine oxidase coupling reaction system.

To assess the sensitivity to oxidative stress, overnight cultures were washed with 0.9% (w/v) NaCl, and resuspended in 0.9% NaCl to a final OD_600_ = 0.2. Fifteen microliters of 30% H_2_O_2_ was added to 1 ml of bacterial suspension. The suspension was held at room temperature for 40 min. Serial dilution of samples in 0.9% NaCl were placed on LB agar with rifampicin and incubated overnight at 28°C. The initial 100% survival point was determined by counting the colony forming unit (CFU) before addition of the H_2_O_2_ [29].

### Comparative proteomic analysis

A detail description of the comparative analysis of cellular proteins of *P*. *chlororaphis* G5 and a *gacA* mutant G5-6 by two-dimensional electrophoresis used in this study is presented in S1 File.

### Extraction and detection of lipopolysaccharides (LPS)

Bacteria were grown in LB with shaking at 28°C for 24 hr. Lipopolysaccharides were extracted using the LPS Extraction Kit (iNtRON Biotechnology, Inc., Japan) according to manufacturer’s instruction. For Tricine-SDS-PAGE, 40μl of 0.25x buffer A [12% (w/v) SDS, 6% (v/v) mercaptoethanol, 30% (w/v) glycerol, 0.05% (w/v) Coomassie Blue G-250, 150 mM Tris/HCl, pH 7.0] was added to LPS pellets with vortex, followed by boiling it for 5 min before loading samples [30]. Protein molecular-weight standards are not presented due to the relative motilities of the LPS do not correlate with those of the protein standard markers [31].

### Statistical analysis

All data were subjected to statistical analysis using SPSS 14.0 (SPSS Inc., USA). ANOVA analysis was conducted with 95% confidence intervals. All experiments were repeated at least twice with three replicates for each treatment.

## Results

### Isolation and identification of a *gacA* mutant of *P*. *chlororaphis* G5

A mini-Tn5Km mutant library of *P*. *chlororaphis* G5 was constructed with a view to identify regulators of phenazine biosynthesis and quorum sensing. From the initial screening, ca. 1000 white mutant colonies with lost pigmentation and hence unable to produce phenazine were cross-streaked against the AHL biosensor strain *C*. *violaceum* CV026 to identify mutants deficient in AHL production. An AHL-negative mutant with lost ability to suppress the pathogenic fungus *R*. *cerealis* (the causative agent of the sharp eyespot disease in cereal) *in vitro* was identified and designated as G5-6. Inverse PCR and sequence analysis of the nucleotide region flanking the transposon revealed that the insertion site of the mini-Tn5 transposon is 105-bp downstream of the response regulator *gacA* (GenBank: FJ969507) translational start site which corresponds to the predicted phosphorylation and intermolecular recognition site of GacA. Multiple sequences alignment demonstrated that the GacA from strain G5 shares 99% identity at the amino acid level to its homologue in *P*. *choloraphis* A23 (GenBank: AEA02287). Introduction of plasmid pUCP26-*gacA* harbouring a functional *gacA* gene in the *gacA* mutant G5-6 restored the production of orange pigment and AHLs, and antifungal activity near to the wild type levels (Fig A in S2 File).

### GacA was essential for AHL accumulation in *P*. *chlororaphis* G5

**Fig 1:**
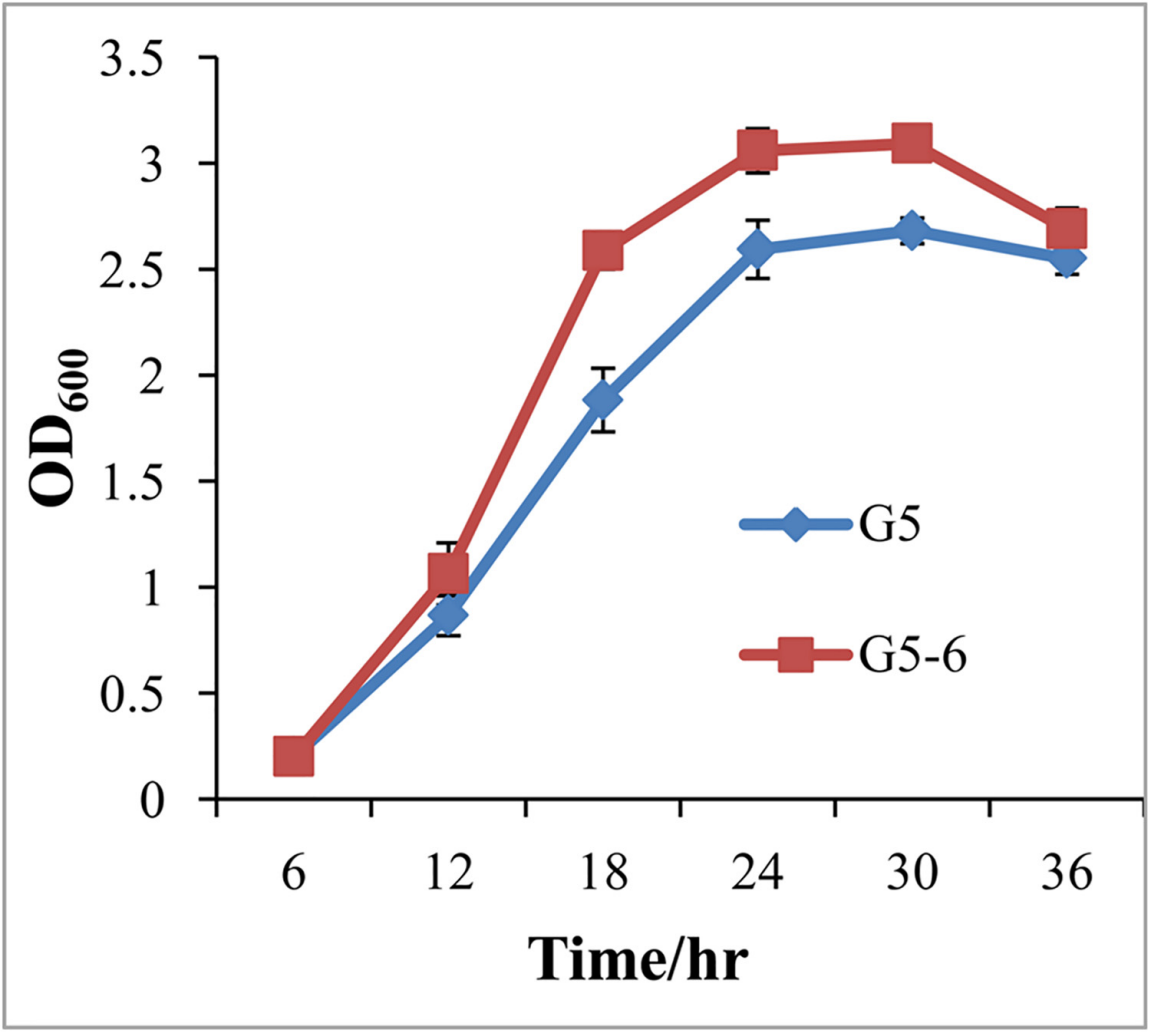
A *gacA* mutation stimulated bacterial growth. Bacterial growth of the wild type G5 and the *gacA* mutant G5-6 was monitored by measuring OD_600_. Overnight cultures of all strains were normalized to an OD_600_ = 0.01, incubated in LB at 28°C with aeration up to 36 hr.

We compared the growth rate of strain G5 and the *gacA* mutant by monitoring OD_600_. Mutation in *gacA* resulted in a marked increase in bacterial growth in *P*. *chlororaphis* G5 (Fig 1), but almost abolished the production of AHL signals as seen using the biosensor *C*. *violaceum* CV026 (Fig A in S2 File), which was further confirmed by LC-MS/MS analysis of the AHL profiles between the G5 parent, the G5-6 mutant and complemented strain. As shown in Table 1, LC-MS/MS analysis showed that G5 can produce at least twelve different AHLs with long- or short-chain in varying abundance including unsubstituted AHLs (C4-HSL, C6-HSL, C8-HSL), 3-oxo derivatives (3-O-C4-HSL, 3-O-C6-HSL, 3-O-C8-HSL, 3-O-C10-HSL) and 3-hydroxy derivatives (3-OH-C4-HSL,3-OH-C6-HSL, 3-OH-C8-HSL, 3-OH-C10-HSL, 3-OH-C12-HSL) exhibiting more diverse AHL profile than what has been described in other *P*. *chlororaphis* strains. The most abundant, and probably more biological relevent AHLs in strain G5 are *N*-(3-oxo-hexanoyl)-L-homoserine lactone (3-O-C6-HSL), *N*-(3-hydroxy-hexanoyl)-L-homoserine lactone (3-OH-C6-HSL), *N*-hexanoyl-L-homoserine lactone (C6-HSL) and *N*-(3-hydroxy-octanoyl)-L-homoserine lactone (3-OH-C8-HSL). *gacA* inactivation resulted in only trace amounts of 3-O-C6-HSL, 3-OH-C6-HSL, 3-OH-C8-HSL and C6-HSL were detected, but complementation with *gacA* expressed from its native promoter almost restored the AHL production to the wild type level indicating that quorum sensing circuits in G5 are highly regulated by the Gac system.

**Table 1:** Relative quantification of AHL production by LC-MS/MS analysis^a^.

Synthetic standards	G5-WT (μM)	G5/pUCP26 (μM)	G5-6/pUCP26 (μM)	G5-6/*gacA* ^*+*^ (μM)
C4-HSL	0.6±0.10	0.73±0.15	0	0.85±0.15
C6-HSL	5.00±0.70	3.02±0.10	0.09±0.01	2.97±0.47
C8-HSL	0.27±0.09	0.13±0.02	0	0.14±0.02
3-oxo-C4-HSL	0.01±0.00	0.01±0.00	0	0.01±0.00
3-oxo-C6-HSL	8.25±1.92	8.64±2.10	0.13±0.03	7.37±3.31
3-oxo-C8-HSL	0.34±0.01	0.21±0.03	0	0.17±0.24
3-oxo-C10-HSL	0.06±0.00	0.02±0.00	0	0.02±0.00
3-OH-C4-HSL	0.04±0.01	0.02±0.01	0	0.02±0.01
3-OH-C6-HSL	7.49±2.16	4.57±0.41	0.08±0.01	5.85±1.73
3-OH-C8-HSL	3.24±0.17	1.57±0.13	0.04±0.01	2.57±0.67
3-OH-C10-HSL	0.44±0.06	0.13±0.03	0	0.15±0.04
3-OH-C12-HSL	0.02±0.00	0	0	0

^a^ A wide range of synthetic AHLs with or without a 3-oxo or 3-hydroxy (OH) substitution, and with even acyl side-chain lengths ranging from C4 to C14 each at either 1 μM or 5 μM concentrations were used as standards. AHLs were identified and confirmed by comparing both the elution time and the MS spectra of the peaks obtained with those of the standards.

### GacA regulation of the production of biocontrol factors and IAA shows some niche specificity

The Gac system has been recognized as a key regulator of biocontrol-related properties in plant-beneficial *Pseudomonas* species [13, 15, 18]. In agreement with previous findings, phenotypic analysis showed that GacA in the endophytic strain G5 positively regulated the production of antibiotics phenazine and HCN (Fig B in S2 File), and protease activity, but negatively controlled siderophore production (Fig C in S2 File).

**Fig 2:**
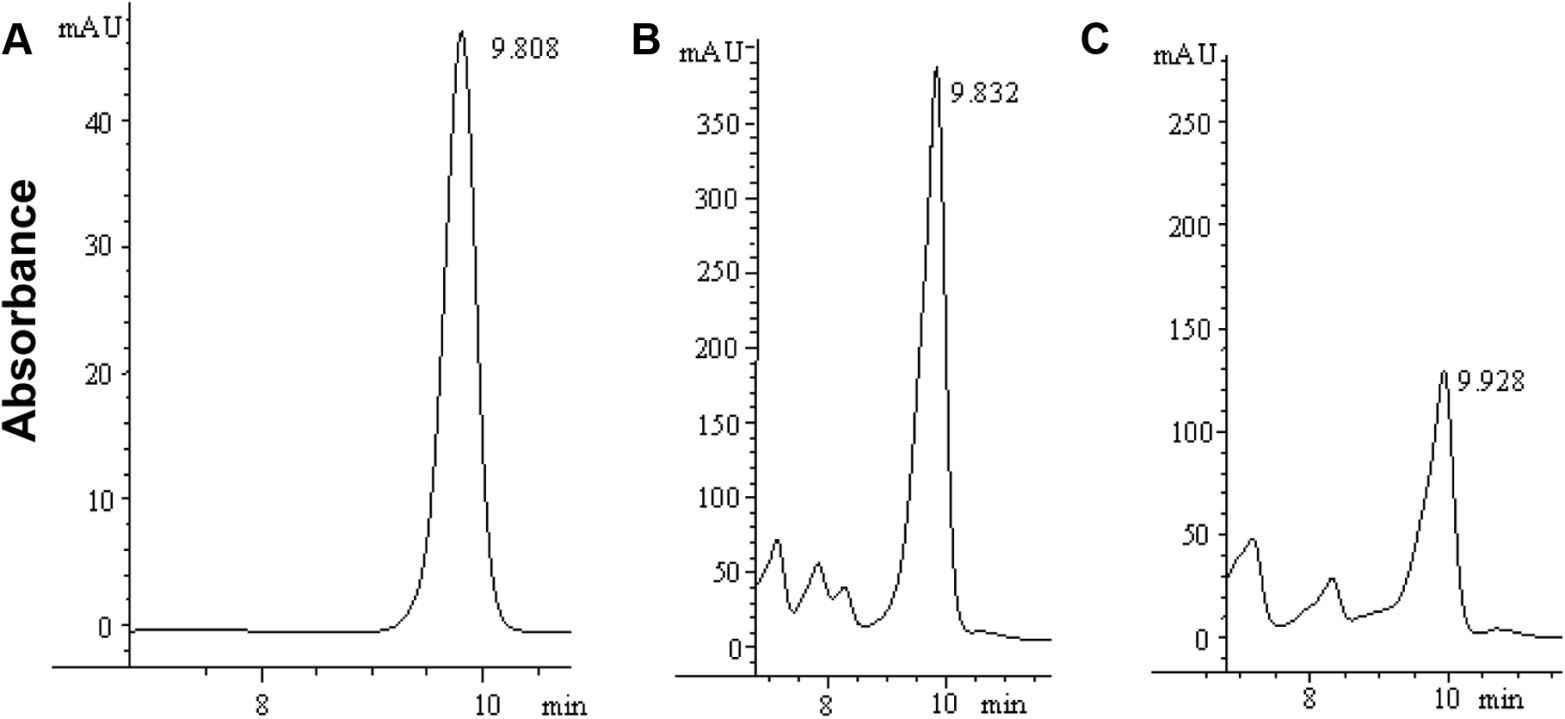
GacA positively controlled IAA biosynthesis. HPLC analysis was performed to detect IAA production with 50 μg/ ml synthetic IAA (A) as a standard between the wild type G5 (B) and the *gacA* mutant G5-6 (C). 20 μl of each sample were loaded and eluted isocratically at a 1 ml/ min flow rate.

In the rhizobacterium *P*. *chlororaphis* O6, GacS has been shown to negatively regulate IAA production [32]. In contrast, HPLC analysis revealed that GacA from the endophytic *P*. *chlororaphis* G5 is a positive regulator of plant auxin IAA (p<0.01). As shown in Fig 2, *gacA* inactivation resulted in about four-fold decreased IAA production suggesting that Gac regulation has an element of niche specificity.

### *gacA* inactivation decreased cell motility, but induced biofilm formation and oxidative stress tolerance

**Fig 3:**
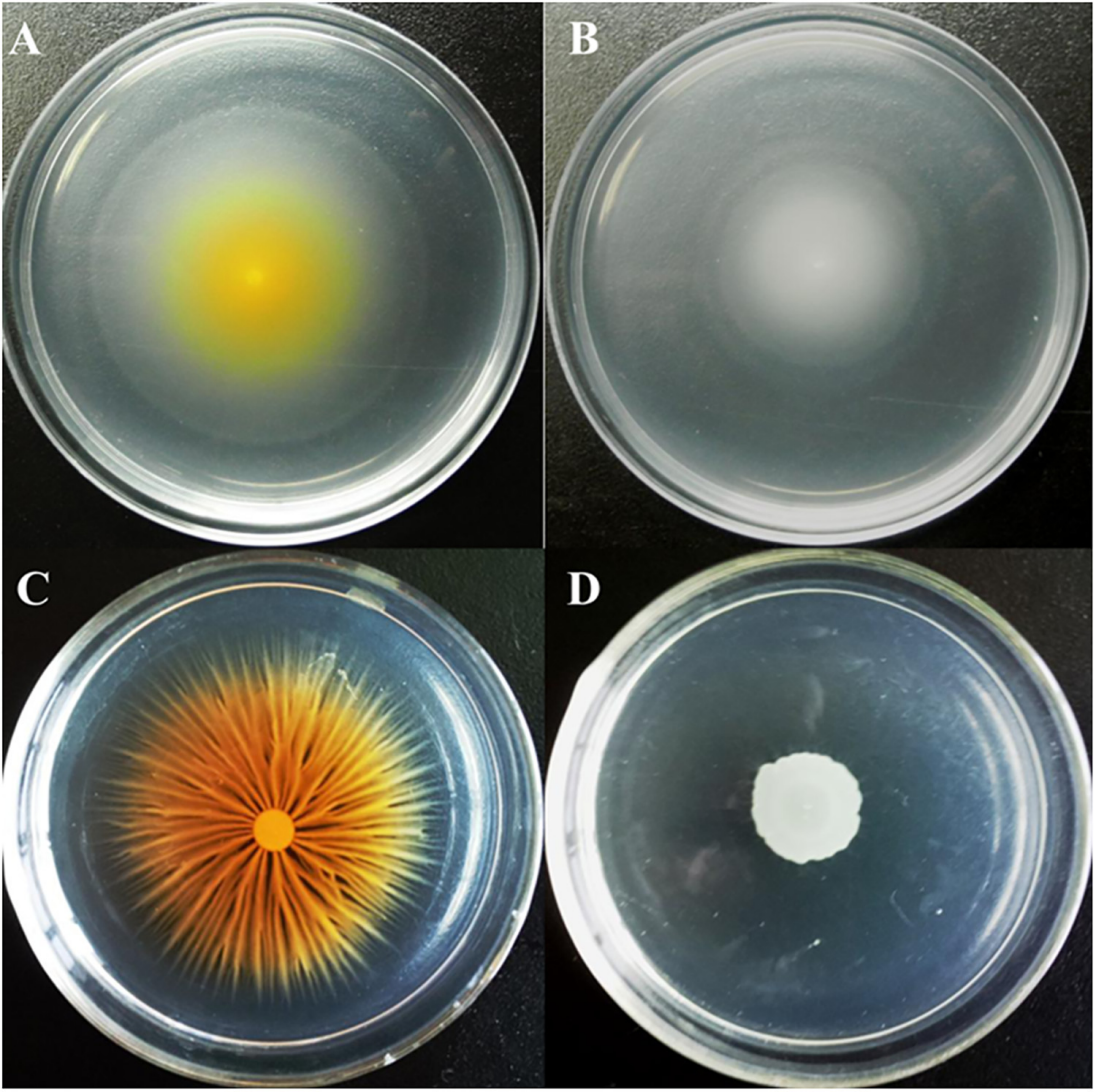
GacA was required for swimming and swarming motility. The wild type G5 (A, C) and the *gacA* mutant G5-6 (B, D) were inoculated on the 0.25% agar swimming plates (top panel) or the 0.4% agar swarming plates (bottom panel), and incubated for 24 hr at room temperature.

The Gac system from *P*. *chlororaphis* has been shown to inhibit cell motility and enhance biofilm formation important for plant colonization and the fitness [13, 17, 19, 33]. To assess whether this regulation was conserved or strain-specific as observed in the control of IAA production, the impact of the GacA mutation in motility and biofilm formation was tested. As shown in Fig 3, in contrast to what has been shown for other *P*. *chlororaphis* strains, the *gacA* mutant greatly decreased the ability to swim (Fig 3B) compared to the wild type (Fig 3A), and a similar trend was observed for swarming motility (Fig 3C and 3D).

**Fig 4:**
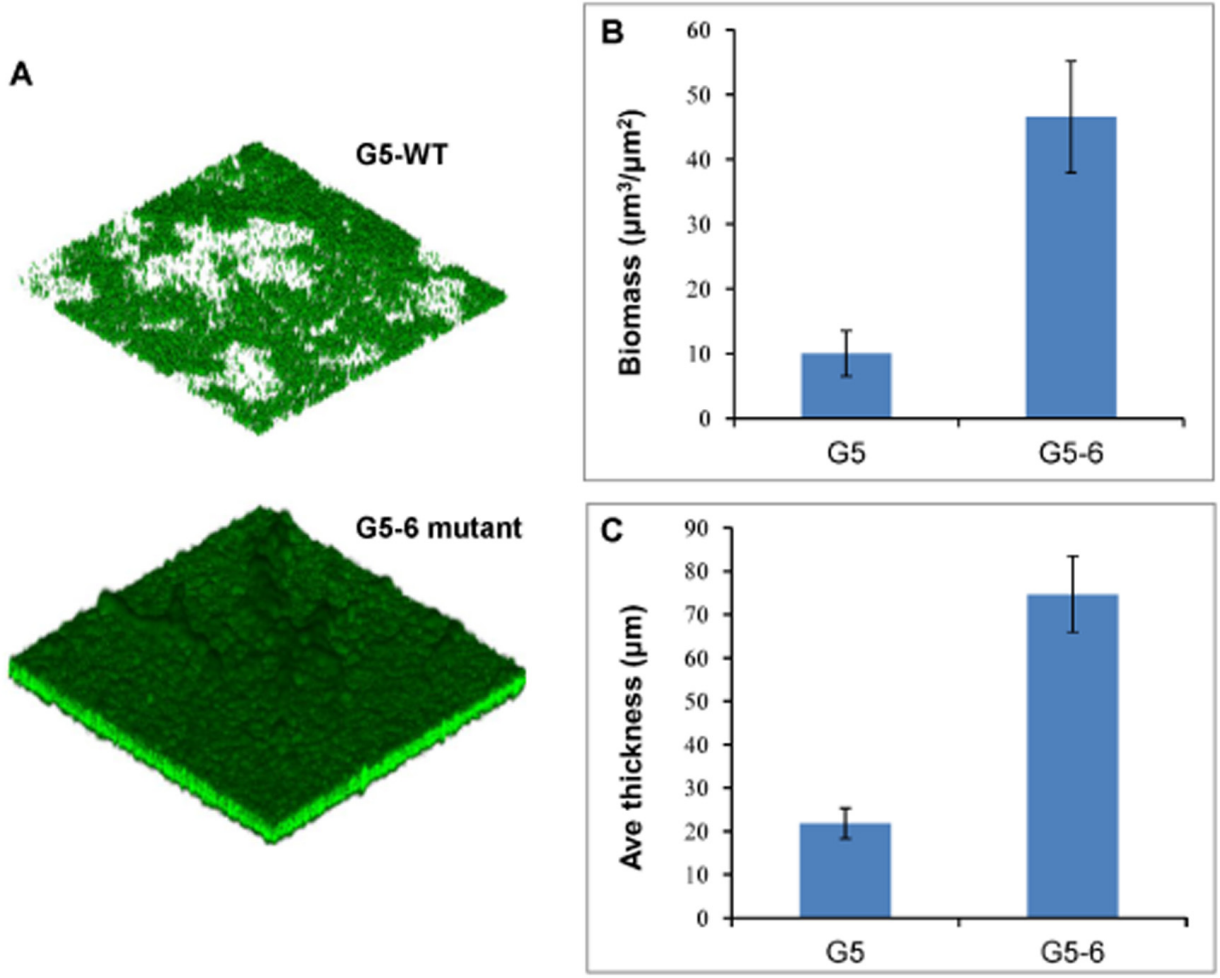
*gacA* inactivation induced biofilm formation. **A,** biofilm formation after 48 hr incubation at 30°C with shaking at 80 rpm was visualized by CLSM. **B,** biofilm biomass and **C,** the average thickness were quantified between the wild type G5 and the *gacA* mutant G5-6 using COMSTAT2 software (p<0.01).

The impact of the *gacA* mutation on biofilm formation was also tested after incubation of bacteria at 30°C for 48 hr using 24-well microtiter plate assays by LCSM observation. As shown in Fig 4A, *gacA* inactivation greatly induced biofilm formation. Further quantitative analysis confirmed that both the biofilm biomass (Fig 4B) and the average thickness (Fig 4C) between these two strains were significantly different. These results support the idea of a strain specificity regulation by the Gac system.

**Fig 5:**
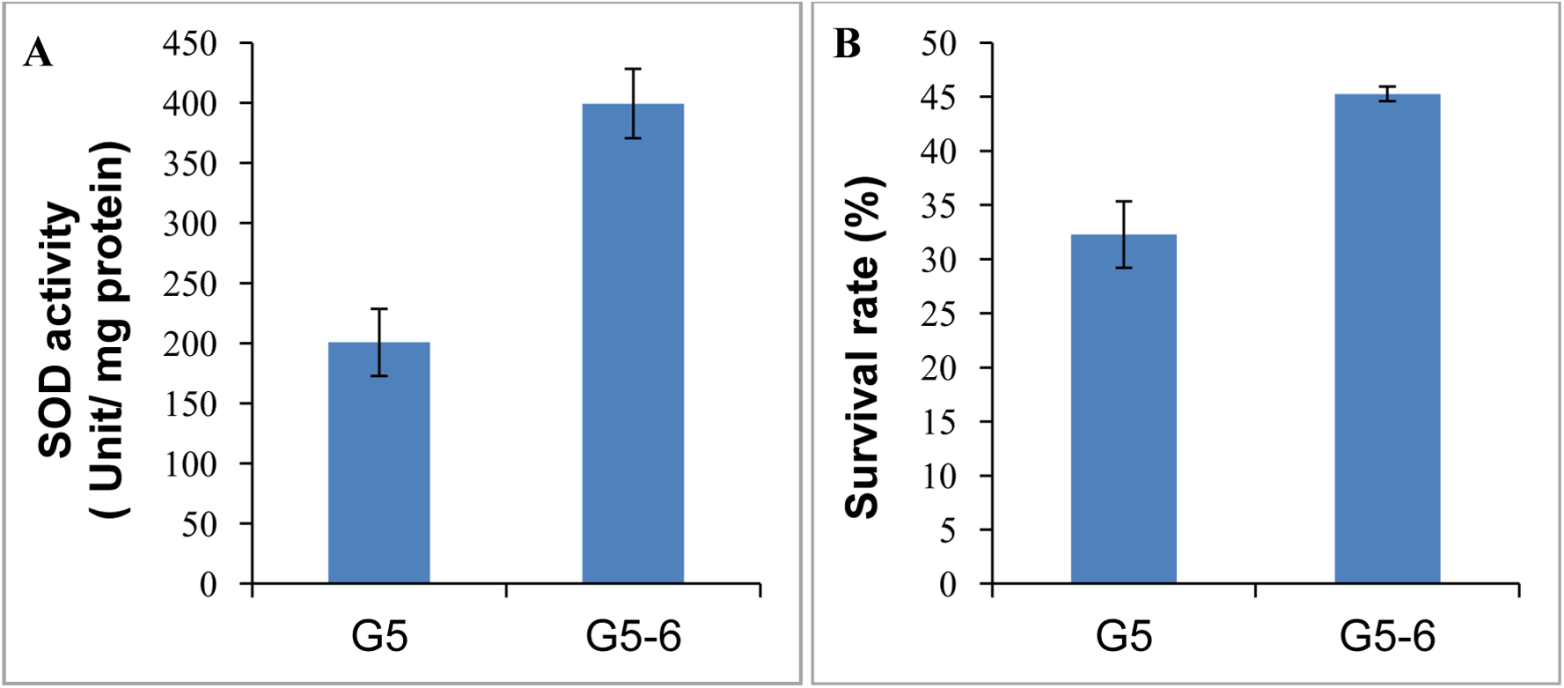
A *gacA* mutation enhanced superoxide dismutase activity and tolerance to oxidative stress. **A,** superoxide dismutase **(**SOD) activity was determined using the Superoxide Dismutase Assay Kit (Suzhou Comin Biotech. Ltd., China). One unit of SOD activity was defined as the amount required to inhibit the rate of reduction of superoxide anion by 50% per microgram of protein (p<0.01). **B,** stationary-phase cultures of the wild type and the *gacA* mutant were assessed for their sensitivity to H_2_O_2_ exposure in 0.9% NaCl for 40 min as survival rate. The initial 100% survival point was determined by counting CFU before addition of the H_2_O_2_ (p<0.05)_._

Excess reactive oxygen species (ROS) or oxidants can damage many cell components, as well as cause disruptions in normal mechanisms of cellular signalling. Antioxidant gene regulation to maintain ROS homeostasis is one of the principal mechanisms of protection against oxidative stress [34]. GacA are known to positively regulate resistance to oxidaive stress in pseudomonads [9, 11, 15]. To determine whether GacA has impact on oxidative stress response in strain G5, we tested the cell sensitivity to hydrogen peroxide and superoxide dismutase (SOD) activity which is essential for oxidative stress response. In contrast to *P*. *chlororaphis* O6 where GacS has been shown to positively regulate the catalase/peroxidase KatG involved in oxidative stress [15], GacA in strain G5 negatively modulated SOD activity and tolerance to H_2_O_2_ exposure (Fig 5).

### Proteomic profiles of *P*. *chlororaphis* G5 and a *gacA* mutant

**Fig 6:**
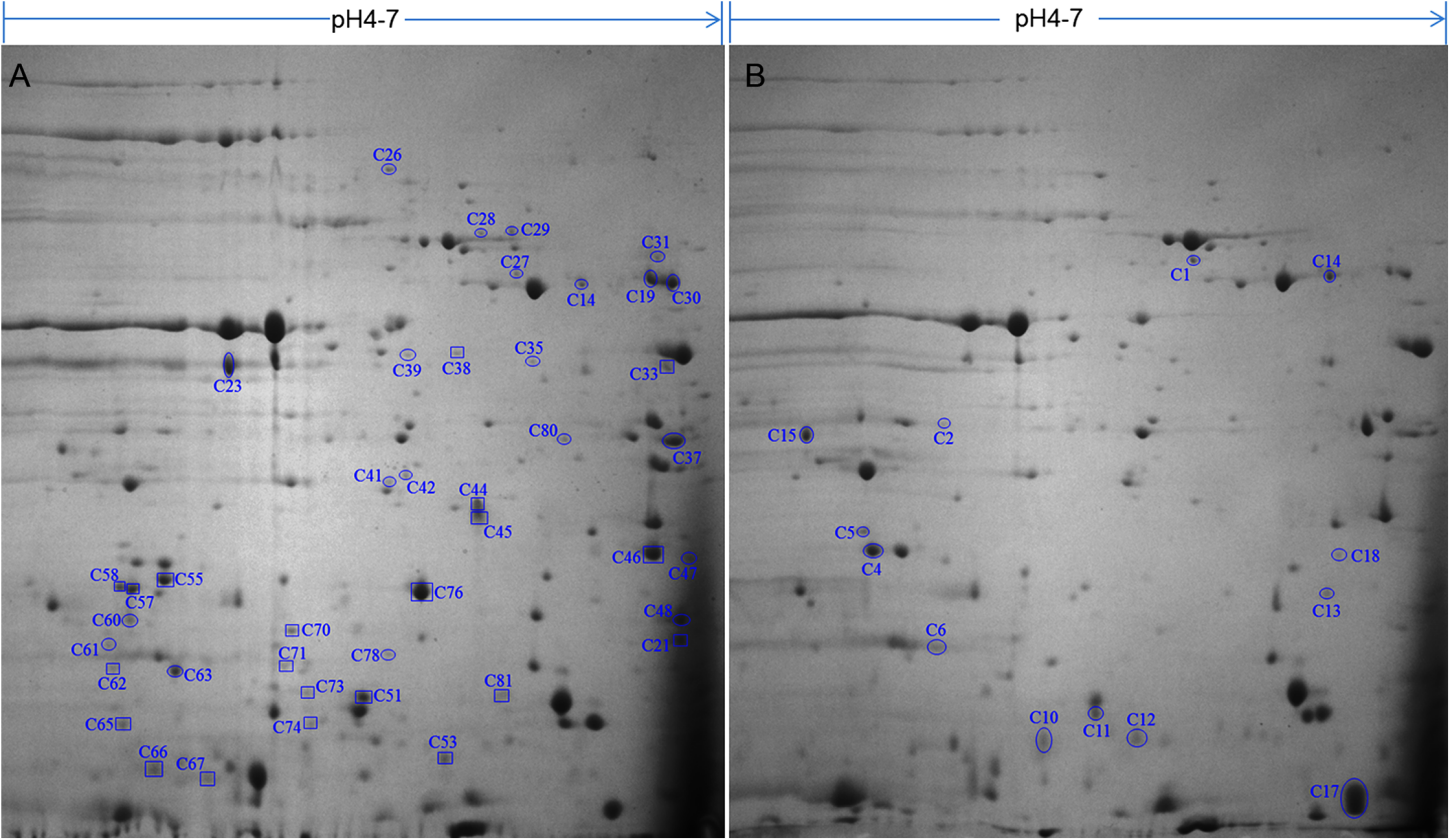
Cellular proteomic profiles of the wild type (A) and the *gacA* mutant (B). A total of 800 μg of each protein sample in triplicate was loaded for IEF, followed by 12% SDS-PAGE. The differentially expressed protein spots (>1.5 fold changes) were numbered (p<0.05). Circle indicates up-regulated spots in WT (A) or in the *gacA* mutant (B), square indicates protein spots only found in the wild type (A).

The results above show that there are marked strain-dependent differences between the impacts of the Gac system in the biology of *P*. *chlororaphis* strains isolated from different niches. To gain a better understanding of global role of GacA in *P*. *chlororaphis* G5, a comparative proteomic analysis of cellular protein extracts from the wild type G5 and the *gacA* mutant G5-6 prepared from early stationary phase cultures (24 hr) was performed, followed by MALDI-TOF Mass Spectrometry (MS) identification. A total of 55 cellular protein spots identified by MS were found to be differentially produced (>1.5 fold; p<0.05) including 13 negatively regulated and 42 positively regulated proteins with 22 spots only found in the wild type G5 (Fig 6, S2 Table). Further bioinformatics analysis using blast2GO revealed that the *gacA*-dependent targets are involved in several distinct biological processes (S2 Table) including primary, secondary metabolisms and energy production; transcription, translation, and signal transduction; membrane biogenesis, transport and secretion; information storage and processing. For example, the abundance of proteins PhzA (C21) and PhzB (C48) encoding the antibiotic phenazine biosynthesis gene clusters was much lower in the *gacA* mutant relative to WT, which agree with the TLC assay for the end product phenazine (Fig B in S2 File) as described above. Correspondingly, *gacA* inactivation resulted in 3.5-fold decreased abundance in the outer membrane efflux protein OprM (C26) contributed to antibiotic transport.

#### GacA had a negative impact on lipopolysaccharide and trehalose biosynthesis and a positive effect on energy metabolism

**Fig 7:**
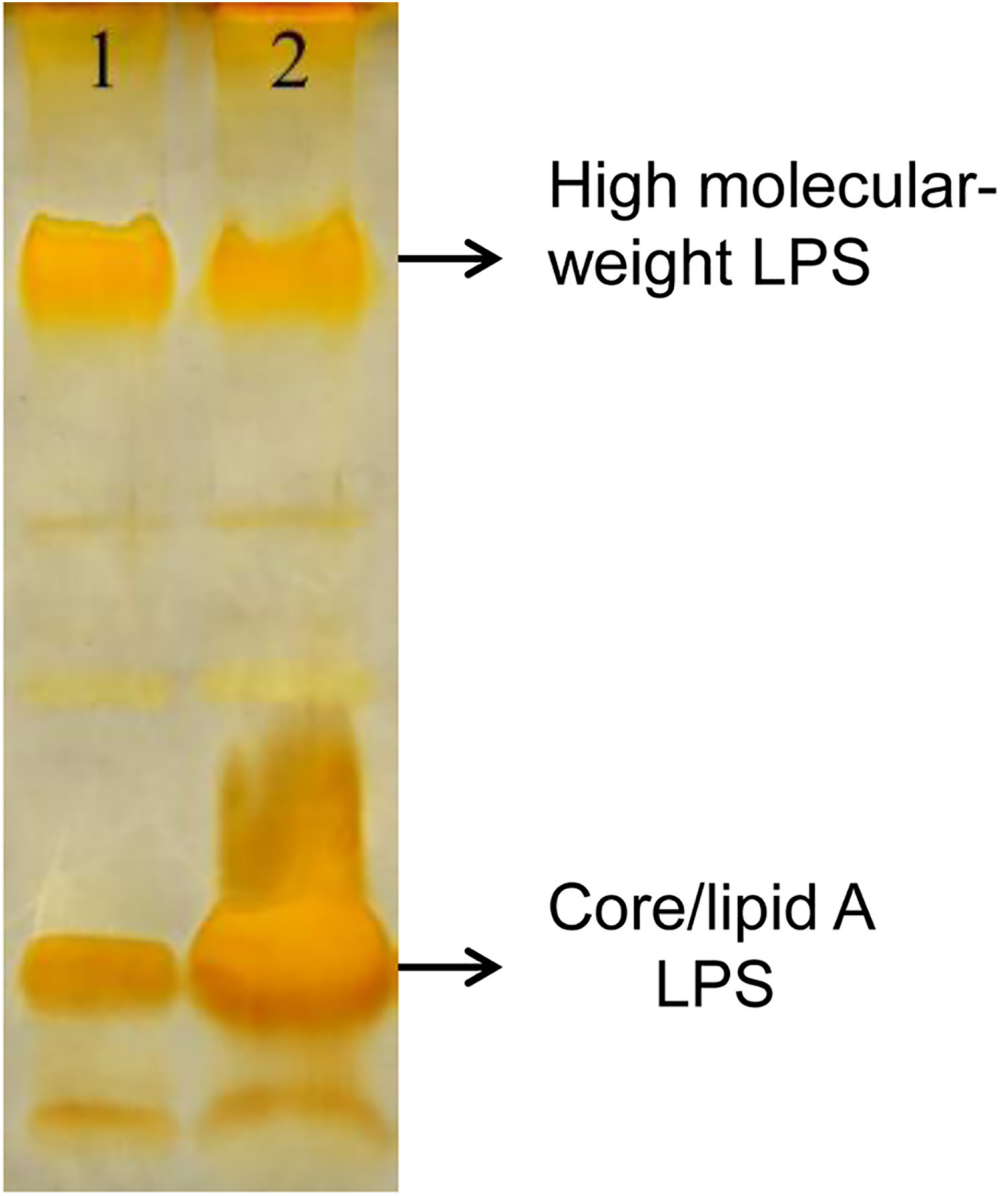
A *gacA* mutation elevated LPS production. Lipopolysaccharides from the wild type G5 and the *gacA* mutant G5-6 were extracted using the LPS Extraction Kit (iNtRON Biotechnol. Inc., Japan), followed by 12% Tricine-SDS-PAGE separation after treatment with proteinase K. Lane 1: the wild type G5; Lane 2; the *gacA* mutant G5-6.

Bacterial polysaccharides including peptidoglycan, lipopolysaccharides (LPS), capsular and exopolysaccharides (EPS) function as structural cell-wall components or important virulence factors enabling bacteria to survive in harsh environments. Therefore polysaccharide biosynthesis is a tightly regulated, energy intensive process [35]. In strain G5 cellular proteome (Fig 6, S2 Table), we found that the LPS biosynthesis protein (C17) and the malto-oligosyltrehalose synthase (C10) responsible for production of the disaccharide trehalose showed increased expression (1.7- and 2.2-fold, respectively) in the *gacA* mutant relative to WT, which was further validated by Tricine SDS-PAGE quantification of LPS production (Fig 7). LPS is composed of lipid A, the core oligosaccharide and the O-antigen polysaccharide sharing the general architecture in members of the family *Enterobacteriaceae*, and constituting an integral part of the outer membrane, thus mediates interactions between the bacterial cell and its surrounding environment [36]. The LPS patterns in Fig 7 showed that only the core/lipid A fraction (Rough-LPS), but not the high-molecular-weight LPS with O-antigen was induced in the *gacA* mutant. In agreement with previous observation in *P*. *aeruginosa* showing the core/lipid A Rough-LPS fraction was more prominent in biofilm than in planktonic cells [36].

In contrast, three energy-associated proteins including the key glycolytic enzyme pyruvate kinase (C41), the succinyl-CoA synthetase (C36) involved in TCA cycle, and F0F1 ATP synthase (C78) for energy production showed lower abundance in the *gacA* mutant relative to WT which agrees with the transcriptomic study in the rhizobacterium *P*. *protegens* Pf-5 where 58 genes function in energy metabolism were down-regulated in the *gacA* mutant [9].

## Discussion

Genome-wide transcriptomic studies have established that the Gac/Rsm system in *Pseudomonas* species may activate numerous downstream transcriptional regulators to cause profound transcriptome changes [8, 9, 13, 14]. In comparison, there have been limited proteomic studies on the impact of Gac system in pseudomonads despites their importance in complementing transcriptomic analysis to determine post-transcriptionally regulated processes which is the key in the regulation by GacS/GacA [7, 37]. Recent proteomic study in the rhizobacterium *P*. *chlororaphis* O6 characterized 12 up-regulated proteins by GacS. For example, TrpE and PrnA responsible for biosynthesis of tryptophan and antibiotic pyrrolnitrin, respectively which were also demonstrated playing a role in induced systemic resistance (ISR) or colonization of plant roots. In addition, KatG encoding a catalase/peroxidase involved in oxidative stress and the general porin OprF are also under the positive control of GacS [15]. Similarly, proteomic analysis in strain G5 showed that PhzA/B responsible for biosynthesis of antibiotic phenazine and the outer membrane protein OprF were also controlled positively by GacA (Fig 6, S2 Table).

In this study, we provided post-transcriptional evidence confirming that GacA may also influence gene transcription in strain G5 through modulating the translation of many intermediate transcriptional factors. In agreement with previous studies [4, 7, 13, 38], AHL profiling confirmed that quorum sensing is *gacA*-dependent. Further proteomic analysis identified many transcriptional regulators differentially expressed by *gacA* inactivation, including LysR-type (C71) and GntR-type (C44) transcriptional factors, the anti-sigma factor MucB (C39) and several TCS regulators like a nitrogen specific histidine kinase NtrB (C67) and a response regulator (C81) (Fig 6, S2 Table) although remain to be reconfirmed. In addition, phenotypic analysis confirmed that GacA in strain G5 was also required for the production of biocontrol factors like antibiotics phenazine, HCN and proteases, consistent with previous observation that the GacS/GacA system functions as a key regulator of secondary metabolisms and extracellular enzymes crucial for biocontrol activity in plant beneficial strains of *Pseudomonas* [13, 15]. However, GacA has also been reported as a negative regulator of phenazine production in *P*. *aeruginosa* M18 [14]. Unexpectedly, in contrast to the rhizobacterium *P*. *chlororaphis* O6 [33], IAA production is positively regulated by the Gac system in the endophytic G5. However, it is worth noting that Salkowski assay of the cultures from G5 and the *gacA* mutant showed opposite results (data not shown) due to the presence of indole compounds unrelated to IAA in bacterial cultures, suggesting that HPLC or LC-MS analysis is essential to confirm IAA production.

LC-MS/MS analysis exhibited an extremely diverse AHL profile by *P*. *cholororaphis* G5 including short-chain unsubstituted-HSLs, as well as 3-hydroxy- HSLs and 3-oxo-HSLs with short- and long-chain. It is different from observation in the wheat rhizospheric *P*. *chlororaphis* 30–84, which has been reported to produce 3-hydroxy-HSLs with chain lengths of 6, 8, and 10 carbons as major species, as well as short-chain C4-HSL and C6-HSL, but 3-oxo-HSLs had not previously been detected in this organism by LC-MS/MS identification [5] highlighting another difference between *P*. *chlororaphis* strains which could have been influenced in part by their original isolation niche. Previous studies have established that the Gac system positively controls the QS machinery via the stimulation of AHL production in some species of *Pseudomonas* [4, 7,13]. For example, the Gac/Rsm system in the pathogenic *P*. *aeruginosa* PAO1 primarily modulates the C4-HSL production, but a *gacA* mutation has little influence on the amount of the long chain 3O-C12-HSL [38]. In addition, the level of AHL production in the *gacS* or *gacA* mutant of *P*. *chlororaphis* 30–84 was ca. 10% of the level of that in the control strain [4]. However, in the endophytic *P*. *chlororaphis* G5, the biosynthesis of both long- and short-chain AHLs is fully dependent on GacA (Table 1).

The Gac system has been demonstrated to differentially regulate cell motility important for plant rhizospheric colonization in biocontrol pseudomonads. For example, GacS/GacA positively regulates cell motility in *P*. *protegens* Pf-5 [9] and *P*. *fluorescens* Pf0-1[39], but negatively controls swimming motility in *P*. *chlororaphis* 30–84 and O6, *P aeruginosa* M18 and *P*. *fluorescens* F113 [13, 14, 33]. Unlike the closely related strains 30–84 and O6, both swimming and swarming motilities in strain G5 were impaired in the *gacA* mutant supporting the idea of *gacA* showing, not only species-specific regulation, but also a strain specificity within *P*. *chlororaphis*.

It has been proposed that there is cross-talk between the mechanisms controlling biofilm formation and the stress response. A variety of environmental and physiological signals that activate the stress response often lead to biofilm formation; in turn, induction of the stress response genes forms part of the cellular adaptation to the biofilm lifestyle [40]. In contrast to the rhizobacteria *P*. *protegens* CHA0 and Pf-5, and *P*. *chlororaphis* O6 where the Gac system positively regulates oxidative stress [9, 11, 15], a *gacA* mutation in *P*. *cholororaphis* G5 enhanced the tolerance to oxidative stress. GacA has also been showed to be required for biofilm formation in many pathogenic or beneficial *Pseudomonas* species [8, 17, 18, 39]. For instance, the Gac system in *P*. *chlororaphis* 30–84 and PA23 positively regulates biofilm formation via stimulating AHL and phenazine synthesis required for biofilm development [17–20]. However, GacA from *P*. *chlororaphis* G5 negatively modulated biofilm development in an AHL- and phenazine- independent pattern as the *gacA* mutant was also deficient in the production of both AHLs and phenazines.

These findings above highlight the strain-specific mechanisms underlying GacA regulation of the distinctive biology of *P*. *chlororaphis* strains with remarkably metabolic and ecological diversity, and likely due to their differences in specific lifestyle (endophytic or rhizospheric), belonging to different subspecies (*P*. *chlororaphis* subsp. *aurantiaca* G5 or *P*. *chlororaphis* subsp. *aureofaciens* 30–84 and O6), or different geographical distribution (from different habitats in two continents). Differences in the regulation of GacS/A also could be caused by horizontal gene transfer from other taxa. In addition, GacS has been demonstrated to differentially regulate biofilm formation in *P*. *cholororaphis* O6 depending on carbon source. Mutation in *gacS* resulted in reduced biofilm formation with sucrose as the major carbon source, but enhanced biofilm formation when mannitol was substituted for sucrose in the defined medium [41]. More importantly, recent comparative genomics studies provide genetic evidence revealing the existence of tremendous genomic diversity within the *P*. *fluorescens* group including *P*. *chlororaphis* 30–84 and O6. Especially, most of the genes contributed to biological control are in the variable regions of the genome, apart from each strain has hundreds of unique genes that could shape their specific lifestyles and distinctive characteristics [3]. However, the precise mechanisms behind the differentially regulation remain to be explored in future.

Proteomic analysis also provided insights into the factors contributed to biofilm development and revealed that a *gacA* mutation stimulated biosynthesis of LPS and the disaccharide trehalose. LPS is the major carbohydrate component of the cell envelope of Gram-negative bacteria, and has been proposed to be involved in bacterial attachment to abiotic surfaces and biofilm formation in the pathogenic *Aggregatibacter actinomycetemcomitans* [42] and *Klebsiella pneumonia* [43]. Whereas trehalose as a universal general stress response metabolite and an osmoprotectant has been considered to play an important role in the formation and development of the microbial biofilm, besides tolerance to stress [44]. In addition, similar to strain O6 [15], the major outer membrane porin OprF (C23) in strain G5 was also upregulated by the Gac system. Recent study has demonstrated that an *oprF* mutation in *P*. *aeruginosa* resulted in increased biofilm formation and the production of Pel exopolysaccharide via the overexpression of the ECF sigma factors AlgU and SigX which increased the c-di-GMP levels, and in turns this intracellular signal upregulated the levels of the small RNA RsmZ suggesting an interplay between OprF and the Gac/Rsm system [45]. Interestingly, the proteomic analysis in strain G5 also showed that GacA positively regulated MucB (C39), the antagonist of AlgU. Several studies in pseudomonads have shown that inactivation of the anti-sigma factors MucB increased the levels of AlgU resulting in constitutive expression of the *alg* operon, involved in biosynthesis of the polysaccharide alginate and LPS which play an important role in the development and maintenance of biofilm formation [46, 47]. However, whether GacA from strain G5 is also involved in control of polysaccharide production via OprF and/or AlgU remains to be elucidated. In contrast, several energy-associated proteins such as the pyruvate kinase (C41) and succinyl-CoA synthetase (C36) showed decreased abundance implying lower glycolytic and TCA cycle activities. These findings are agreement with the observation in *Staphylococcus* species where the TCA cycle acts as a signalling pathway negatively modulating polysaccharides intercellular adhesins (PIA) biosynthesis and biofilm formation [48, 49]. However, whether the TCA cycle can act as a signal transduction pathway to modulate biofilm development in other bacteria remains to be explored. These results above suggested that the Gac system in *P*. *chlororaphis* G5 may repress biofilm development at least partially through differential regulation of biosynthesis of polysaccharides, trehalose and the energy production to balance out the carbohydrate metabolism. This highlights the impact of proteomic analysis as a powerful tool to gain an insight into regulatory networks accounting for post-transcriptional regulation.

Spontaneous *gac* mutants arising with high frequency in laboratory cultures, and in soil and rhizosphere have been observed in many pseudomonads which suggested the existence of a positive selection for the loss of Gac and likely due to a reduced metabolic load relative to that of the Gac^+^ population [39]. For example, *gac* mutants often present growth advantages with larger colonies and hyper-fluorescence indicating overproduction of siderophores to increase iron acquisition. These phenotypes were also observed in *P*. *chlororaphis* G5 (Fig 1, Fig C in S2 File). On the other hand, Gac^-^ strains are likely to utilize exogenous public goods such as AHL signal molecules and antibiotics secreted by the Gac^+^ neighbour in heterogeneous populations. Consequently, Gac^+^ and Gac^-^ populations can be mutualistic in mix populations [13, 39]. Therefore, the negative-regulated traits by GacA are of particular interest. Apart from growth rate and siderophores, biofilm development and the oxidative stress tolerance were also found to be greatly induced in the *gacA* mutant of strain G5, which is a complete contrast to previous observations in many pseudomonads where GacS/GacA is required for biofilm formation, and the oxidative stress response via RpoS [7–9, 11, 18, 39].

In conclusion, the impact of the Gac system in *P*. *cholororaphis* shows a strain specificity which may be associated with a niche-dependency. The Gac-dependent activation of the quorum sensing regulatory circuits, and the tightly regulation of a large variety of secondary metabolites such as antibiotics phenazine and HCN in *P*. *chororaphis* G5 is an energy intensive process. We hypothesize that GacA inactivation lowered the metabolic burden to allow overproduction of polysaccharides and trehalose important to promote biofilm formation. Furthermore, *gacA* mutants elevated tolerance to oxidative stress, and siderophore production which probably contributed to stress tolerance, competitive adaptation for better colonization and survival of heterogeneous populations in adverse environment. Whether *gacA* mutants somehow confer selective advantages to the mixed populations of *P*. *chlororaphis* G5 still need to be verified in field experiments which may help developing new strategies for the exploitation of mixed populations such as Gac^+^ and Gac^-^ strains in the future management of plant diseases.

## Supporting Information

S1 File Methods for 2D-PAGE and MALDI-TOF/MS analysis. (PDF)

S2 File Phenotypic analysis of the production of AHLs, antibiotics, protease and siderophores, and antifungal activity by strain G5 and its derivatives. (PDF)

S3 File Original MALDI-TOF/MS data. (ZIP)

S1 Table Strains and plasmids used in this study. (PDF)

S2 Table Identification of cellular proteins with altered abundance (> 1.5-fold) in a *gacA* mutant G5-6 relative to G5-WT. (DOCX)
