# Supplementary material for: Regulation of GacA in Pseudomonas chlororaphis Strains Shows a Niche Specificity
Source: PLoS One. 2015 Sep 17;10(9):e0137553. doi: 10.1371/journal.pone.0137553 (PMC4574860; doi:10.1371/journal.pone.0137553)
Supplement: S1 File — (PDF) [file pone.0137553.s001.pdf]

Method S1 for 2D-PAGE and MALDI-TOF/MS analysis

Comparative proteomic analysis of cellular proteins of P. chlororaphis G5 and a gacA mutant G5-6 was conducted by two-dimensional electrophoresis.

For protein extraction, bacteria were grown overnight in LB broth with shaking at 200 rpm at 28°C to entry into the early stationary phase (OD_{600} ~2.0). Cell pellets obtained after centrifugation at 16,000 x *g* for 20 min at 4°C were washed twice with 0.9% (w/v) NaCl, resuspended with 150 mM Tris-HCl (pH 7.5) and disrupted by sonication. Fractions of cellular proteins were precipitated with 10% (w/v) TCA (trichloroacetic acid), followed by centrifugation at 12,000 x *g* for 30 min at 4°C. Protein samples were washed three times using pre-cold acetone, then freeze-dried and stored at -80 C° for further analysis. The protein concentration of each sample was quantified according to Bradford protein assay protocol (Bradford, 1976).

2-D electrophoresis and Image analysis

A total of 800 μg of each protein sample in triplicate was added to rehydration solution (7M Urea, 4% CHAPS (give full name), 2M thiourea, 65mM DTT, 2% ampholine pH 3.5-10) to a final volume of 400μL for passive rehydration for 12h at room temperature. The proteins were focused in the first dimension using 17cm, pH 4-7 IPG strips (Bio-Rad). Isoelectric focusing was then performed using a protein IEF Cell (Bio-Rad) at a constant temperature of 20°C with the program as follows: 200V, 1h; 500V, 1h; 1000V, 1h; 8000V, 5h; and 8000V up to 60000 V h. The gel strips were equilibrated according to the manufacture. The second dimension was performed for protein isolation with 12% polyacrylamide gels (0.7mm thickness) using an **Ettan DALTsix** Vertical Electrophoresis System (GE Healthcare). Gels were run with a constant power of 5 W per gel. Broad molecular weight protein standards (Bio-Rad) were used for molecular weight determination. The gels were stained with Coomassie Brilliant blue R-250 and scanned with a high precision scanner (ScanMaker

9700XL, Microtek). PDQuest 8.01 software (Bio-Rad) was used for gel image analysis to detect the protein expression levels. Normalization was performed for quantity analysis. Student's t-test was performed to determine if the relative change was statistically significant ($P< 0.05$) (Jun *et al*., 2012). Proteins fold change >2.0 for the secretome or fold change >1.5 for the cellular proteome were subjected to further identification by MS (MALDI-TOF/MS?) analysis.

Protein identification by mass spectrometry

Selected protein spots were excised manually from the 2-D gels and digested with trypsin (Promega, USA) for identification by MALDI-TOF/MS. The external calibration of Mass accuracy for peptide mass fingerprint (PMF) analysis was performed with a peptide standard range of 700-4000 Da. The internal calibration was carried out with enzyme autolysis peaks. The raw spectra generated by MS was processed with FlexAnalysis software 2.4 (Bruker Daltonics) with SNAP algorithm. Database searching using the Mascot search engine V2.2 (Matrix Science, UK) against the NCBInr database. Significance threshold was $P<0.05$ (Zhu *et al*., 2011).

In silico **analysis and protein classification**

MS data were retrieved using UniProt database (http://www.uniprot.org/), the sequences were submitted to Gene Ontology (GO) for annotation using the Blast2GO algorithm (Conesa*et al*., 2008). Proteins were categorized into clusters according to their biological function and cell process. The signal peptide and the protein subcellular localization were predicted using signalP 4.1 server (http://www.cbs.dtu.dk/services/SignalP/), and Cello (http://cello.life.nctu.edu.tw/) software, respectively.

References

Bradford MM. A rapid and sensitive method for the quantitation of microgram quantities of protein utilizing the principle of protein-dye binding. Anal Biochem. 1976, 72:248–254.

Conesa A, Gotz S. Blast2GO: A comprehensive suite for functional analysis in plant genomics. International journal of plant genomics. 2008;2008:619832. doi: 10.1155/2008/619832. PubMed PMID: 18483572; PubMed Central PMCID: PMC2375974.

Jun H, Kieselbach T, Jonsson LJ. Comparative proteome analysis of Saccharomyces cerevisiae: A global overview of in vivo targets of the yeast activator protein 1. BMC genomics. 2012;13. PubMed PMID: WOS:000307947300001.

Zhu L, Hu W, Liu D, Tian W, Yu G, Liu X, et al. A reference proteomic database of Lactobacillus plantarum CMCC-P0002. PloS one. 2011;6(10):e25596.
