# Supplementary material for: Regulation of GacA in Pseudomonas chlororaphis Strains Shows a Niche Specificity
Source: PLoS One. 2015 Sep 17;10(9):e0137553. doi: 10.1371/journal.pone.0137553 (PMC4574860; doi:10.1371/journal.pone.0137553)

S2 File Phenotypic analysis of the production of AHLs, antibiotics, protease and siderophores, and antifungal activity by strain G5 and its derivatives

Figure A GacA is required for the production of orange pigment, AHL signals and antifungal activity.

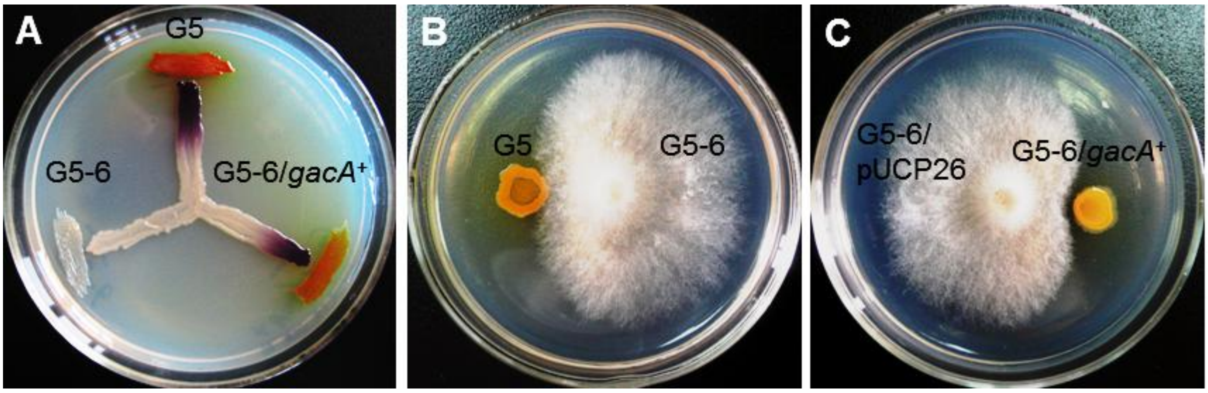

The *gacA* mutant G5-6 is deficient in the production of orange pigment (A), AHLs detected through cross-streak against *C. violaceum* CV026 (A, changed into purple), and the capacity to suppress the fungal pathogen *R. cerealis* (B, C) *in vitro* compared with the wild type G5. Complementation of the *gacA* mutant G5-6 with a plasmid pUCP26 carrying *gacA* expressed from its own promoter (G5-6/*gacA*$^{+}$) restored these phenotypes to nearly wild type levels.

Figure B GacA positively regulates the production of phenazine (A) and HCN (B)

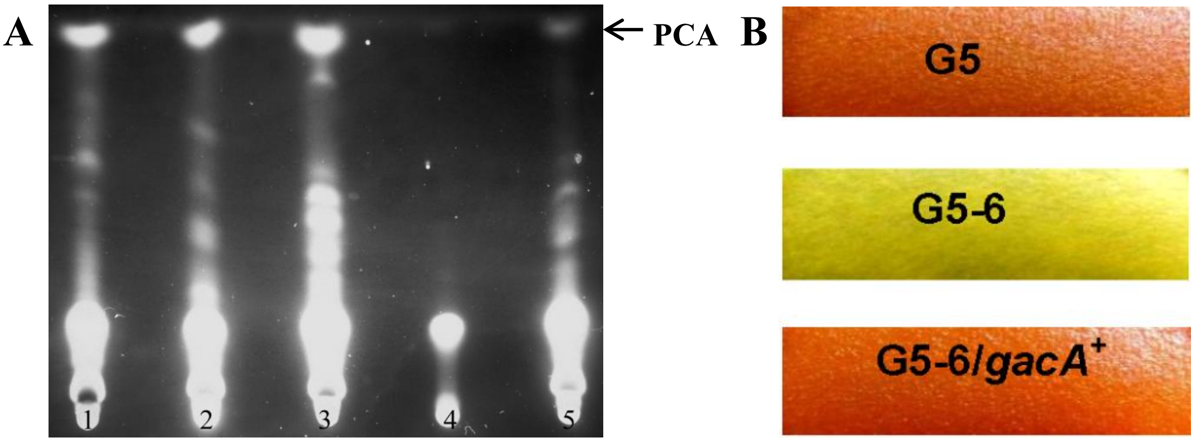

A: The antibiotic phenazine-1-carboxylic acid (PCA) was detected by TLC.

10 μl of each extract were loaded onto the thin-layer Silica Gel 60 F254 plate (Merck, Germany). Lanes 1 & 2: *P. chlororaphis* strains 30-84 and 449 as positive control,

respectively; Lane 3: G5-WT; Lane 4: the *gacA* mutant G5-6; Lane 5: the complemented strain G5-6/*gacA*$^{+}$.

B: HCN was assayed as follows: overnight cultures of strain G5 and derivatives were spread on LB agar containing glycine (4.5 mg/ mL). Sterilized filter paper saturated with 1% solution of picric acid and 2% sodium carbonate was placed in the lid of the petri dish. The petri dish was then sealed and incubated at 28 °C for 4 days. A change in colour of the filter paper from yellow to reddish brown as an indication of cyanogenic activity was recorded.

Figure C A *gacA* mutation resulted in reduced protease activity (A), but overproduced siderophore (B) in the *gacA* mutant G5-6 relative to the wild type G5

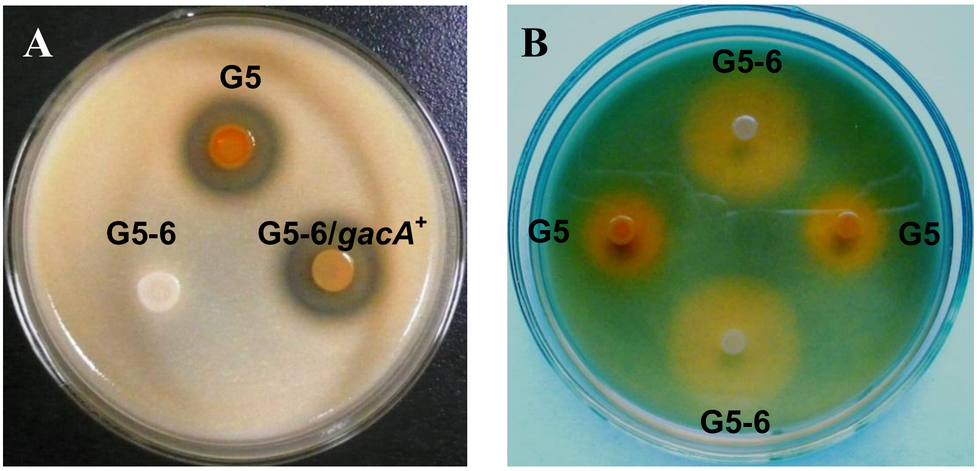

Supplement: S2 File — (PDF) [file pone.0137553.s002.pdf]
