# Supplementary material for: Regulation of GacA in Pseudomonas chlororaphis Strains Shows a Niche Specificity
Source: PLoS One. 2015 Sep 17;10(9):e0137553. doi: 10.1371/journal.pone.0137553 (PMC4574860; doi:10.1371/journal.pone.0137553)
Supplement: S1 Table — (PDF) [file pone.0137553.s004.pdf]

Table S1 Strains and plasmids used in this study

Strain or plasmid	Description	Reference/ source
P. chlororaphis		
G5	Phz^{+},Rif^{r}, wild-type strain isolated from Chinese parsley	Li et al. 2009
G5-6	*gacA*::Tn5 mutant of G5, Km^{r}	This study
G5-6/*gacA*+	G5-6 carrying pUCP26-*gacA* for complementation, Rif^{r}, Tc^{r}	This study
30-84	Phz+,Rif+, wild-type strain isolated from wheat	Maddula et al. 2006
449	Phz+,Rif+, wild-type strain isolated from maize	Veselova et al., 2008
Escherichia coli		
DH5α	F^{-}*endA1 hsdR17* (rK - mK^{+}) *supE44 thi-1 recA1*	Green and Sambrook 2012
	gyrA96 relA1 Φ80d*lacZ*ΔM15 λ^{-}	
S17-1	*thi pro hsdR hsdM recA rpsL*RP4-2 (Tc^{r}::Mu) (Km^{r}::Tn*7*)	Green and Sambrook 2012
C. violaceum CV026	Violacein production-based acyl-HSL bioreporter,Km^{r}	McClean et al. 1997
Plasmids		
pUT mini-Tn5 Km	MiniTn5 transposon, Km^{r}	de Lorenzo and Timmis 1994
pUCP26	Broad host vector, Tc^{r}	West et al, 1994
Rhizoctonia cerealis	Pathogenic fungus, causes sharp eyespot in cereal	Lab collection

References

de Lorenzo V and Timmis KN (1994) Analysis and construction of stable phenotypes in gram-negative bacteria with Tn5- and Tn10-derived minitransposons. Meth Enzymol 235: 386–405.

Green MR, Sambrook J. Molecular cloning : a laboratory manual. Cold Spring Harbor, N.Y: Cold Spring Harbor Laboratory Press; 2012.

Li H, Liu X, Gao K, Jia J. Cloning of *phzIR* from the endophytic *Pseudomonas* sp. G5 and its expression in Escherichia coli. Chin J Biotech. 2009;25:832-9.

Maddula VSRK, Zhang Z, Pierson EA and Pierson III LS. (2006) Quorum Sensing and phenazines are involved in biofilm formation by *Pseudomonas chlororaphis (aureofaciens*) strain 30-84. *Microbiol Ecol.* 52:289-301.

McClean KH, Winson MK, Fish L, Taylor A, Chabra SR, Camara M. *et al.*, (1997) Quorum sensing in Chromobacterium violaceum: exploitation of violacein production and inhibition for the detection of N-acylhomoserine lactones. *Microbiol* 143:3703–3711.

Veselova Ma, Klein Sh, Bass IA, Lipasova VA, Metlitskaia AZ, Ovadis MI *et al*.. Quorum sensing systems of regulation, synthesis of phenazine antibiotics, and antifungal activity in rhizospheric bacterium pseudomonas chlororaphis 449. Russ J Genet 2008, 44 (12):1400-1408.

West SE, Schweizer HP, Dall C, Sample AK & Runyen-Janecky LJ (1994) Construction of improved Escherichia–Pseudomonas shuttle vectors derived from pUC18/19 and sequence of the region required for their replication in Pseudomonas aeruginosa. Gene 148: 81–86.
