# Supplementary material for: Regulation of GacA in Pseudomonas chlororaphis Strains Shows a Niche Specificity
Source: PLoS One. 2015 Sep 17;10(9):e0137553. doi: 10.1371/journal.pone.0137553 (PMC4574860; doi:10.1371/journal.pone.0137553)
Supplement: S2 Table — (DOCX) [file pone.0137553.s005.docx]

**Table S2** Identification of cellular proteins with altered abundance (> 1.5-fold) in a *gacA* mutant G5-6 relative to G5-WT

| **Spot**  **No.** | **Description** | **Acession**  **No.** | **pI/MW**  **(KD)** | **Scores** | **Seq cov**  **(%)^a^** | **Fold^b^** | **SignalP ^c^** | **Subcelluar**  **location^d^** |
| --- | --- | --- | --- | --- | --- | --- | --- | --- |
| **Carbohydrate metabolism and Energy production (tricarboxylic acid cycle and glycolysis)** | | | | | | | | |
| C17 | lipopolysaccharide biosynthesis protein | gi\|238064604 | 6.39/40.56 | 82 | 38 | +1.7 |  | C |
| C10 | malto-oligosyltrehalose synthase | [gi\|302554967](http://www.ncbi.nlm.nih.gov/blast/Blast.cgi?ALIGNMENTS=50&ALIGNMENT_VIEW=Pairwise&AUTO_FORMAT=Semiauto&CDD_SEARCH=on&CLIENT=web&COMPOSITION_BASED_STATISTICS=on&DATABASE=nr&DESCRIPTIONS=100&ENTREZ_QUERY=(none)&EXPECT=10&FILTER=L&FORMAT_BLOCK_ON_RESPAGE=None&FORMAT_OBJECT=Alignment&FORMAT_TYPE=HTML&GAPCOSTS=11+1&I_THRESH=0.001&LAYOUT=TwoWindows&MATRIX_NAME=BLOSUM62&NCBI_GI=on&PAGE=Proteins&PROGRAM=blastp&QUERY=MREGTNGGGAGTYVSMTPERTDPVVPTATYRLQLQPDFPFGAAAAVVPYLASLGVSHLHLSPVLEAVPGSAHGYDVVDHARVREELGGEEGLRALARTAREHGLGLVADIVPNHMAMAPRHNRALWEVLREGPKSPYARWFDIDWEAQDGQVLLPVLGGPLGEVLGELRVDGDVLRYYDHAFPLREGTGDLPLPHLLDAQWYRPVWWRLARTELNYRRFFSISELIGVRVEDPEVFEATHGKILQLLHEGVIDGLRVDHPDGLADPDGYLRRLHEATGGRWTVVEKILADGERLPASWPVAGTTGYDALRHVDGLFTDPAGFGDLLGQYRRFAAPQTDRGGHWEATVRRAAYKVLTHELATETDRLTRVAARLCATSPEPALRDRAPWALRTALQELLVRMEVYRPYESVDAASVVTEEAAAEARLAFAVPEEAGAVDVVRDLVLGRYGDGPAQVEFRTRFAQTSSALRAKSVEDTAFYRYVPLLSATEVGGDPGRPALSPEEFHAYCARVQRDWPVTGTVVSTHDTKRSADVRAALNVLTECPDRWADVLAEVTRTGEGVPDAQLAWAAWQTVFGLGPADAERVRGALLKHVREAGLYTSWTEQEAPYEEAVARFVTAGPCGTPGERVASFRDALEPHIRANVLGTALVQLTMPGVPDVYQGTEAEYRALVDPDNRRAVGFPPEVSGATSEEKLTVTRAALGLRARHPGAFGDTATYTPLTAEGPAARHCLAFVRSGTALTAVTRLSLRLEEAGGWQGTRLPLPPGRWADVLDPGREFTGHARVADLLGRSPVALLERVGE&SERVICE=plain&SET_DEFAULTS.x=9&SET_DEFAULTS.y=5&SHOW_OVERVIEW=on&WORD_SIZE=3&END_OF_HTTPGET=Yes) | 5.46/87.76 | 84 | 17 | +2.2 |  | C |
| C41 | pyruvate kinase/ glycolysis | gi\|187933683 | 5.44/50.83 | 81 | 22 | -2.3 |  | C |
| C63 | succinic semialdehyde dehydrogenase/TCA | gi\|289704612 | 4.57/ 49.23 | 82 | 31 | -5.8 |  | C |
| **Amino acid, nucleotide metabolism** | | | | | | | | |
| C4 | 2,3,4,5-tetrahydropyridine-2-carboxylate N-succinyltransferase, DapD | [gi\|154148079](http://www.ncbi.nlm.nih.gov/blast/Blast.cgi?ALIGNMENTS=50&ALIGNMENT_VIEW=Pairwise&AUTO_FORMAT=Semiauto&CDD_SEARCH=on&CLIENT=web&COMPOSITION_BASED_STATISTICS=on&DATABASE=nr&DESCRIPTIONS=100&ENTREZ_QUERY=(none)&EXPECT=10&FILTER=L&FORMAT_BLOCK_ON_RESPAGE=None&FORMAT_OBJECT=Alignment&FORMAT_TYPE=HTML&GAPCOSTS=11+1&I_THRESH=0.001&LAYOUT=TwoWindows&MATRIX_NAME=BLOSUM62&NCBI_GI=on&PAGE=Proteins&PROGRAM=blastp&QUERY=MKIQNLDELKNFTDEIRSKNGYKDPFIWAIGRQSVGSFGKKTIKMDYAEINLKSNYASAAILINAAIKNGTKIDFSDSELIVPLNKDIIGYALKKMNFLIGEANGEKHKNLQILMQIDKTLKENEKFTKYISHKFCVVFIFDDVAPKSVESVYLKLYALSKNLTAPRSLNLNGAFGVLPNLAWDIYGEPVELEMLRKDEISNKFGCSKNCAKIAYVDKFPRFLAHIIPENNIRILDDSKVRLGAAIAPGTTIMPGAAYVNFNAGTLGSAMVEGRISSSVIVGEGSDIGGGASILGVLSGTNGNPISIGNHCLLGANSVTGIPLGDDCIVDAGIAVLEGTKILMDEKNRAELAKLNPNFDFSKEIYKGLELANLNGLHFRQNSQNGQMTVSLSKRAIKLNKDLH&SERVICE=plain&SET_DEFAULTS.x=9&SET_DEFAULTS.y=5&SHOW_OVERVIEW=on&WORD_SIZE=3&END_OF_HTTPGET=Yes) | 8.71/44.27 | 82 | 28 | +2.0 |  | O,C |
| C5 | bifunctional OHCU decarboxylase | [gi\|222110249](http://www.ncbi.nlm.nih.gov/blast/Blast.cgi?ALIGNMENTS=50&ALIGNMENT_VIEW=Pairwise&AUTO_FORMAT=Semiauto&CDD_SEARCH=on&CLIENT=web&COMPOSITION_BASED_STATISTICS=on&DATABASE=nr&DESCRIPTIONS=100&ENTREZ_QUERY=(none)&EXPECT=10&FILTER=L&FORMAT_BLOCK_ON_RESPAGE=None&FORMAT_OBJECT=Alignment&FORMAT_TYPE=HTML&GAPCOSTS=11+1&I_THRESH=0.001&LAYOUT=TwoWindows&MATRIX_NAME=BLOSUM62&NCBI_GI=on&PAGE=Proteins&PROGRAM=blastp&QUERY=MAWTLEQLNAASPAEALQMLDGLYEHSPWIAEAALQQRPFRSLAQLKYAMVQVLNGAGLERLLELIRAHPELAGKAMVAQALTTESTNEQSQAGLTHCTPEEFARIQQLNADYNARFGFPFILAVRGPRGTGLSRQEIIATFARRLSNHPDFERAEALRNIHRIAEIRLNDKFGAEPVLGNDVWDWHERLAQHSDPGYAEKGQLTVTYLTDAHRACAQRISHWMRDCGFDEVEIDAVGNVVGRYKPATPDGKYLMTGSHYDTVRNGGKYDGRLGIFVPMACVRELHRQARRLPFGIEVVAFAEEEGQRYKATFLGSGALIGDFRNEWLDQKDADGITMREAMQHAGLCIGDIPKLRRDAARYLGFIEVHIEQGPVLNELDIPLGVVTSINGGVRYVCEMLGMASHAGTTPMDRRRDAALGVAELALYMEQRAARDGDSVATIGMLQVPGGSINVVPGKCSFSLDMRAPTDAQRDAMVADVLAQLEEIAQRRGLQYRAELAMKVAAAPSAPAWQHRWEKAVDALGVPVYRMPSGAGHDAMKLHEIMAQAMLFVRGLHGGISHNPLESSTADDIQLAVDAFSHVLEQLAAE&SERVICE=plain&SET_DEFAULTS.x=9&SET_DEFAULTS.y=5&SHOW_OVERVIEW=on&WORD_SIZE=3&END_OF_HTTPGET=Yes) | 5.76/65.26 | 88 | 29 | +1.8 |  | C |
| C28 | aspartate-semialdehyde dehydrogenase | gi\|307726603 | 5.43/40.46 | 81 | 30 | -3.2 |  | P,C |
| C19 | uridylate kinase, PyrH | gi\|227821899 | 7.60/30.41 | 97 | 37 | -3.2 |  | I,C |
| C51 | aspartate carbamoyltransferase, PyrB | gi\|254474169 | 8.83/31.37 | 83 | 50 | W |  | C |
| **Lipid/ fatty acid and inorganic ion metabolism** | | | | | | | | |
| C31 | acetyl-CoA acetyltransferase | gi\|77458301 | 5.74/40.80 | 87 | 22 | -3.1 |  | C |
| C75 | polyphosphate kinase, ppk | gi\|298345563 | 6.15/92.53 | 81 | 17 | W |  | C |
| C62 | putative manganese-dependent inorganic pyrophosphatase | gi\|255654392 | 4.89/59.64 | 86 | 31 | W |  | C |
| **Secondary metabolites and transport** | | | | | | | | |
| C21 | phenazine biosynthesis protein, PhzA | gi\|62822973 | 6.65/18.83 | 96 | 76 | W |  | C |
| C48 | phenazine biosynthesis protein, PhzB | gi\|62822974 | 6.52/19.03 | 141 | 73 | -3.2 |  | C |
| C26 | outer membrane efflux protein/ response to antibiotic/ drug transmembrane transport, OprM | gi\|70734041 | 5.86/52.67 | 240 | 53 | -3.5 | + | O |
| **Membrane/envelope biogenesis and transport** | | | | | | | | |
| C11 | Oligopeptide ABC ATP binding cassette transporter | gi\|242373148 | 5.92/38.15 | 86 | 31 | +2.6 |  | C |
| C23 | Porin OprF/ion transporter, OprF | gi\|4580967 | 5.44/34.81 | 106 | 67 | -3.3 | + | O |
| C60 | peptidoglycan-associated outer membrane lipoprotein,OprL | gi\|259090537 | 5.22/16.59 | 92 | 52 | -4.2 | + | O |
| C78 | F0F1 ATP synthase subunit B, AtpD | gi\|77461951 | 5.77/17.02 | 84 | 39 | -2.3 |  | P,C |
| C73 | phosphate ABC transporter, PstB | gi\|148263584 | 9.49/36.62 | 86 | 32 | W | + | P |
| C46 | lipid ABC transporter permease, MsbA | gi\|116074681 | 10.10/32.15 | 82 | 43 | W |  | O,P |
| **Protein secretion systems, effector, chaperon and stress response** | | | | | | | | |
| C12 | type II secretion system protein C, GspC | gi\|300973518 | 9.60/30.96 | 81 | 37 | +5.1 |  | P |
| C18 | FKBP-type peptidyl-prolyl cis-trans isomerase B, FlkB | gi\|70731273 | 5.87/18.26 | 83 | 54 | +1.9 |  | C |
| C15 | GrpE protein | gi\|77456989 | 4.59/20.85 | 140 | 60 | +1.6 |  | C |
| C29 | Adhesion | gi\|282853946 | 4.93/48.36 | 82 | 31 | -6.2 |  | C |
| C74 | lpxtg-motif cell wall anchor domain protein | gi\|225867339 | 5.33/365.18 | 103 | 6 | W | + | O,P |
| C38 | Esx-1 secretion-associated protein, EspL | gi\|15611016 | 4.73/12.19 | 89 | 51 | W |  | C |
| **Information storage and processing (DNA replication, recombination, repair and transcription )** | | | | | | | | |
| C2 | DNA polymerase III subunits gamma and tau, DnaX | [gi\|114568986](http://www.ncbi.nlm.nih.gov/blast/Blast.cgi?ALIGNMENTS=50&ALIGNMENT_VIEW=Pairwise&AUTO_FORMAT=Semiauto&CDD_SEARCH=on&CLIENT=web&COMPOSITION_BASED_STATISTICS=on&DATABASE=nr&DESCRIPTIONS=100&ENTREZ_QUERY=(none)&EXPECT=10&FILTER=L&FORMAT_BLOCK_ON_RESPAGE=None&FORMAT_OBJECT=Alignment&FORMAT_TYPE=HTML&GAPCOSTS=11+1&I_THRESH=0.001&LAYOUT=TwoWindows&MATRIX_NAME=BLOSUM62&NCBI_GI=on&PAGE=Proteins&PROGRAM=blastp&QUERY=MDDTPLPAEDTGATEALMPGLDLPATGPTPGYQVLARKYRPDTFEDLIGQDAMVRTLTNAFAAGRIAHAYMLTGVRGVGKTTTARLIARALNFQTDGIDAPSMALGEKGRHCDAIGRSAHVDVMEMDAASRTGVGDIREILEGVRYAPVSARYKVYIIDEVHMLSTSAFNALLKTLEEPPEHAKFIFATTEIRKVPVTVLSRCQRFDLKRIDREVLTDHLDRICGLEGASVERDGLSLIARAAEGSVRDALSLLDQAIVQGSDEDGPVSAPQIRDMLGLADHARVLDLLEQTLTGKTADALAELSSLHDAGGDPVVITRDLLDYVHATARVKAAGPGADLGEAADTVARIVTLAEGQTLGQLTRMWKILLTGLDDVRNAPDALAAAEMTVLRLASAASLPPPEDAARLLAGMPRATSPASGAGEAPGKPEPAPASATGSASAAMDRDAAAHRALSAPEAVAQVEQDAPAISGPQTWEALMDLLREKRDIGLQSDVERYVRPAVFKPGSFTFQPIEDAPRDLAQRLSRRLLEWTGERWMILADGSLEGGETWSERRLRLKAERLEAARHDPAVTEVMRLFPGAEIVKIRDPQPTAETDTDATTEKRA&SERVICE=plain&SET_DEFAULTS.x=9&SET_DEFAULTS.y=5&SHOW_OVERVIEW=on&WORD_SIZE=3&END_OF_HTTPGET=Yes) | 5.01/65.13 | 85 | 23 | +1.7 |  | C |
| C80 | replication initiator protein | gi\|152973767 | 8.75/32.52 | 91 | 40 | -6.8 |  | O,C |
| C22 | DNA primase, DnaG | gi\|310816222 | 6.51/74.19 | 89 | 19 | -4.4 |  | C |
| C66 | DNA primase, DnaG | gi\|312110190 | 6.87/68.87 | 82 | 16 | W |  | C |
| C35 | exonuclease III, RecD | gi\|229591315 | 5.77/31.00 | 84 | 22 | -1.6 |  | C |
| C56 | exodeoxyribonuclease V, gamma subunit, RecC | gi\|116328992 | 6.54/131.87 | 82 | 21 | W |  | O |
| C61 | IS30 family transposase | gi\|311063532 | 11.38/34.03 | 86 | 43 | -1.9 |  | C |
| C76 | transposase | gi\|85705574 | 10.88/13.96 | 91 | 47 | W |  | P |
| C47 | putative nucleotide-binding protein, YajQ | gi\|70732100 | 6.774/18.2 | 105 | 45 | -1.9 |  | C |
| **Translation** | | | | | | | | |
| C13 | 16S rRNA methyltransferase | gi\|212637843 | 5.41/23.63 | 88 | 49 | +3.8 |  | C |
| C1 | elongation factor Tu, | [gi\|70732884](http://www.ncbi.nlm.nih.gov/blast/Blast.cgi?ALIGNMENTS=50&ALIGNMENT_VIEW=Pairwise&AUTO_FORMAT=Semiauto&CDD_SEARCH=on&CLIENT=web&COMPOSITION_BASED_STATISTICS=on&DATABASE=nr&DESCRIPTIONS=100&ENTREZ_QUERY=(none)&EXPECT=10&FILTER=L&FORMAT_BLOCK_ON_RESPAGE=None&FORMAT_OBJECT=Alignment&FORMAT_TYPE=HTML&GAPCOSTS=11+1&I_THRESH=0.001&LAYOUT=TwoWindows&MATRIX_NAME=BLOSUM62&NCBI_GI=on&PAGE=Proteins&PROGRAM=blastp&QUERY=MAKEKFERNKPHVNVGTIGHVDHGKTTLTAALTRVCSEVFGSARVDFDKIDSAPEEKARGITINTAHVEYDSNIRHYAHVDCPGHADYVKNMITGAAQMDGAILVCSAADGPMPQTREHILLSRQVGVPYIVVFLNKADMVDDAELLELVEMEVRDLLSTYDFPGDDTPIIIGSALMALNGQDDNEMGTTAVKRLVETLDTYIPEPERAIDKPFLMPIEDVFSISGRGTVVTGRVERGIVRIQEEVEIVGLRDTQKTTCTGVEMFRKLLDEGRAGENCGVLLRGTKRDDVERGQVLVKPGTVKPHTKFTAEVYVLSKEEGGRHTPFFKGYRPQFYFRTTDVTGNCELPEGVEMVMPGDNIQMTVTLIKTIAMEDGLRFAIREGGRTVGAGVVAKVIE&SERVICE=plain&SET_DEFAULTS.x=9&SET_DEFAULTS.y=5&SHOW_OVERVIEW=on&WORD_SIZE=3&END_OF_HTTPGET=Yes) | 5.24/44.03 | 138 | 37 | +1.9 |  | C |
| C6 | 30S ribosomal protein S6 | [gi\|70734081](http://www.ncbi.nlm.nih.gov/blast/Blast.cgi?ALIGNMENTS=50&ALIGNMENT_VIEW=Pairwise&AUTO_FORMAT=Semiauto&CDD_SEARCH=on&CLIENT=web&COMPOSITION_BASED_STATISTICS=on&DATABASE=nr&DESCRIPTIONS=100&ENTREZ_QUERY=(none)&EXPECT=10&FILTER=L&FORMAT_BLOCK_ON_RESPAGE=None&FORMAT_OBJECT=Alignment&FORMAT_TYPE=HTML&GAPCOSTS=11+1&I_THRESH=0.001&LAYOUT=TwoWindows&MATRIX_NAME=BLOSUM62&NCBI_GI=on&PAGE=Proteins&PROGRAM=blastp&QUERY=MRHYEIIFLVHPDQSEQVGGMVERYTKLIEEDGGKIHRLEDWGRRQLAYAINNVHKAHYVMLNVECTGKALAELEDNFRYNDAVIRNLVIRREEAITGQSEMLKAEENRSERRERRDRPEHADSADGDDSDNSDASDNADE&SERVICE=plain&SET_DEFAULTS.x=9&SET_DEFAULTS.y=5&SHOW_OVERVIEW=on&WORD_SIZE=3&END_OF_HTTPGET=Yes) | 4.93/16.40 | 166 | 71 | +3.0 |  | C |
| C42 | methionine-tRNA ligase | gi\|138893709 | 5.67/74.88 | 89 | 23 | -2.1 |  | C |
| **Transcrational regulator and signal transduction mechanisms** | | | | | | | | |
| C14 | protein kinase, PknB | gi\|162457201 | 5.99/143.41 | 96 | 21 | +2.4 |  | C |
| C39 | sigma factor algU regulatory protein, MucB | gi\|70728829 | 5.92/35.16 | 105 | 31 | -1.7 | + | C |
| C44 | transcriptional repressor, GntR family | gi\|288574504 | 7.85/25.73 | 83 | 48 | W |  | C |
| C53 | RNA polymerase ECF-type sigma factor | gi\|150002717 | 6.21/23.77 | 88 | 44 | W |  |  |
| C71 | aminoethylphosphonate catabolism associated LysR family transcriptional regulator | gi\|319784965 | 6.68/31.90 | 91 | 28 | W |  | C |
| C67 | signal transduction histidine kinase, nitrogen specific NtrB | gi\|307292785 | 6.84/39.87 | 84 | 24 | W |  | C |
| C81 | two-component regulatory system response regulator protein CitB | gi\|206564594 | 25.87/6.67 | 85 | 51 | W |  | C |
| **Protein of unknown function** | | | | | | | | |
| C27 | LmbE family protein | gi\|255531282 | 8.40/89.12 | 86 | 17 | -2.6 | + | P |
| C37 | hypothetical protein | gi\|312621177 | 9.36/12.04 | 84 | 73 | -2.0 |  | C |
| C45 | hypothetical protein DSY0869,YqeB | gi\|89893615 | 6.77/28.58 | 85 | 49 | W |  | C |
| C55 | hypothetical protein Pfl01_5595 | gi\|77461816 | 5.00/18.20 | 182 | 45 | W |  | C |
| C58 | hypothetical protein Pfl01_5595 | gi\|77461816 | 5.00/18.20 | 141 | 55 | W |  | C |
| C65 | hypothetical protein Pfl01_5595 | gi\|77461816 | 5.00/18.20 | 156 | 56 | W |  | C |
| C57 | hypothetical protein PSPA7_3615 | gi\|152988603 | 4.99/18.29 | 134 | 52 | W |  | C |

*55 protein spots were identified by MALDI-TOF MS analysis , 13 proteins are upregulated (+), 42 proteins are downreulated (-) in the *gacA* mutant relative to WT including 22 spots only found in the wild type (W= square in Figure 7)

a: Sequence coverage; b: Fold change in protein abundance of the gacA mutant relative to WT; c: Signal peptide prediction,”+” represents protein with SP sequence.

d: Subcellular localization prediction, cytoplasmic(C), Periplasmic (P), Outer membrane (O), Inner membrane (I).
